# HPLC-FLD determination of aflatoxins M_1_ and M_2_ in raw cow milk samples using in-syringe gas-controlled density tunable solidification of a floating organic droplet-based dispersive liquid–liquid microextraction method†

**DOI:** 10.1039/d3ra04149b

**Published:** 2024-02-08

**Authors:** Maede Rabie, Mohammadhosein Movassaghghazani, Mohammad Reza Afshar Mogaddam

**Affiliations:** a Faculty of Veterinary Medicine, Shabestar Branch, Islamic Azad University Shabestar Iran; b Department of Food Hygiene and Quality Control, Faculty of Veterinary Medicine, Shabestar Branch, Islamic Azad University Shabestar Iran mh.movassagh@iau.ac.ir drmhmg@gmail.com +98-9143010292; c Food and Drug Safety Research Center, Tabriz University of Medical Sciences Tabriz Iran; d Pharmaceutical Analysis Research Center, Tabriz University of Medical Sciences Tabriz Iran

## Abstract

Herein, an in-syringe gas-controlled density tunable solidification of a floating organic droplet-based dispersive liquid–liquid microextraction method was employed for the extraction of aflatoxin M_1_ and M_2_ from cow milk samples prior to their quantification with high-performance liquid chromatography equipped with a fluorescence detector. In this method, after precipitating the proteins of the sample using a zinc sulfate solution, the supernatant phase was transferred into a barrel of a glass syringe, with the end closed with a septum containing a mixture of menthol, phenylacetic acid DES (as the extraction solvent), and chloroform (as a density modifier). After that, an inert gas was bubbled into the syringe. In this manner, chloroform was evaporated and fine droplets of extractant were released, which extracted the analytes during their passing. Finally, the syringe was placed in an ice bath and the obtained solidified drop was injected into the separation system after diluting with a mobile phase. Under the best analysis conditions, low limits of detection (1.45 and 1.86 ng L^−1^ for AFM_1_ and AFM_2_, respectively) and quantification (4.83 and 6.21 ng L^−1^ for AFM_1_ and AFM_2_, respectively), high extraction recovery (75 and 70% for AFM_1_ and AFM_2_, respectively), and good precision (relative standard deviations ≤ 4.8%) were obtained by employing the approach reported in this study. In the end, this method was successfully employed to determine AFM_1_ and AFM_2_ in raw cow milk samples collected from Tabriz, Iran.

## Abstract 2

An in-syringe gas-controlled density tunable solidification of a floating organic droplet-based dispersive liquid–liquid microextraction method was employed for the extraction of aflatoxins from cow milk samples prior to their quantification with HPLC equipped with a fluorescence detector.

## Graphical abstract

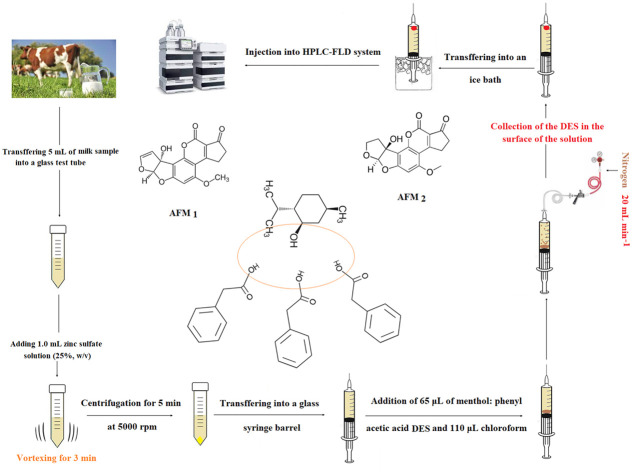

## 1. Introduction

Aflatoxins (AFs) are toxic and carcinogenic substances that appear in foodstuffs due to the fungal growth of *Aspergillus flavus* and *Aspergillus parasiticus* species during their production, packaging, transportation, and storage.^1^ AFB_1_, AFB_2_, AFG_1_, AFG_2_, AFM_1_, and AFM_2_ are the six most important and toxic (the International Agency for Research on Cancer classified these AFs as group I carcinogens) members of these compounds.^2^AFM_1_ and AFM_2_ are highly toxic AFs that are known as major derivatives of AFB_1_ and AFB_2_, respectively (after ingestion, AFB_1_ and AFB_2_ are hydroxylated and metabolized in the cow's liver and subsequently excreted in milk, urine, and meat).^3^ The intake of AFM_1_ and AFM_2_ through diet and their accumulation in the human body can lead to mental disorders, immunotoxicity, chronic toxicity, carcinogenic toxicity, and mutagenicity.^4^ Therefore, monitoring AFM_1_ and AFM_2_ in foodstuffs such as cow milk, which is nutrient-rich (containing various vitamins, carbohydrates, proteins, and minerals), and extensively utilized (especially highly consumed by infants and elderly people who are unable to digest solid food well) is of great importance.^5–7^ The European Union has established maximum residual limits (MRLs) for AFM_1_ in milk samples (50 ng kg^−1^) to guarantee their safety.^8^ However, up to now, no MRL has been set for AFM_2_ in milk. According to the literature, high-performance liquid chromatography (HPLC) with a fluorescence detector (FLD) and tandem mass spectrometry (MS/MS) are the most commonly employed analytical instruments for the determination of AFs such as AFM_1_ and AFM_2_ in various samples.^9,10^ It should be mentioned that FLD is preferred due to its low cost in comparison with HPLC-MS/MS. Commonly, because of the complex matrices of food samples, *e.g.*, milk samples (the presence of high percentages of proteins and fatty acids can restrict the extraction of analytes) and low concentration of analyte residues in them, an efficient sample preparation process before their instrumental analysis is required.^11^ Liquid–liquid extraction (LLE) is a traditional method for the extraction of analytes into the appropriate organic solvent.^12^ To overcome the problems of LLE (consuming high volumes of toxic organic solvents and being tedious), researchers are looking for alternative methods; as a result of these efforts, liquid phase microextraction (LPME) based methods are being introduced.^13^ Solidification of floating organic droplet-based dispersive liquid–liquid microextraction (SFOD-DLLME) is the well-known and efficient model of LPME.^14^ In this method, initially, a mixture of extraction (with low melting point and lower density compared to water) and dispersive solvents is injected into the sample solution and placed in a test tube. Thus, the fine droplets of the extractant are formed and the analytes are extracted into them. The test tube is then placed in an ice bath to help accumulate and solidify the droplets.^15^ Subsequently, the droplet is transferred to a microtube using a spatula and allowed to melt. Considering the above procedure, SFOD-DLLME is faster (owing to the removal of the centrifuging process, which is a time-consuming step) and safer (because of using greener solvents, *e.g.* 1-undecanol instead of toxic halogenated solvents) than conventional DLLME.^16,17^ Generally, the ease of operation and providing high extraction recovery (ER) and enrichment factor (EF) are considered as the major advantages of SFOD-DLLME.^18^ In recent years, several SFOD-DLLME-based works have been reported using new generation of green solvents, *e.g.*, deep eutectic solvents (DESs) and ionic liquids.^19,20^ DESs are prepared through a reaction between a hydrogen-bond donor (HBD) and a hydrogen-bond acceptor (HBA) at an elevated temperature.^21^ The melting point of the formed solvent is lower than that of its components.^22^

The key aim of this research was to validate and apply an in-syringe gas-controlled density tunable SFOD-DLLME method for the efficient extraction of AFM_1_ and AFM_2_ from cow milk samples. The extracted analytes were determined using HPLC-FLD. Acceptable extraction times, high ERs, simplicity, and good repeatability are the chief merits of the offered method. According to our preliminary studies, this is the first report on the application of the offered approach for the extraction and quantification of AFM_1_ and AFM_2_ in raw cow milk samples.

## 2. Experimental

### 2.1. Materials and solutions

AFM_1_ and AFM_2_ standards were purchased from Sigma-Aldrich (St. Louis, MA, USA). Acetonitrile (ACN), HPLC-grade water, NaCl, menthol, phenylacetic acid, zinc sulfate, and choline chloride (ChCl) were purchased from Merck (Darmstadt, Germany). A stock solution of the AFs (at a concentration of 10 mg L^−1^ of each analyte) was prepared in ACN and utilized in the validation and optimization steps.

### 2.2. Synthesis of DESs

DESs were prepared based on a previously published procedure.^23^ Therefore, in menthol : phenylacetic acid and ChCl : phenylacetic acid DESs, phenylacetic acid (as an HBD) was mixed with menthol and ChCl (as HBAs) at molar ratios of 1 : 3 and 1 : 1 in two tubes. Subsequently, the tubes were heated in a water bath maintained at 60 °C for an hour.

### 2.3. Raw cow milk samples

Twenty-one raw cow milk samples were obtained from local producers (Tabriz, East Azarbaijan Province, Iran). After primary studies, one of these samples that were free of AFs (this point was confirmed by a validated analytical method^24^) was taken and utilized as a blank. All the samples were maintained in a refrigerator at 4 °C before their analysis by the suggested method.

### 2.4. Instruments

In this work, an Agilent Liquid Chromatograph (Model 1200) equipped with an FLD was utilized for the quantification of AFM_1_ and AFM_2_. A Luna C_18_ ODS (2) column (150 × 4.6 mm, 3 μm particle size) (Phenomenex, Torrance, CA, USA) maintained at 30 °C was employed to isolate AFM_1_ and AFM_2_. For separation of the analytes, an isocratic elution using ACN and water (25 : 75, v/v) was used at a flow rate of 1 mL min^−1^ for 15 min. For FLD, 365 and 435 nm were set as the excitation and emission wavelengths, respectively. All injections were performed utilizing a 20 μL sample loop. A Labinco L46 vortex mixer (Breda, Netherlands) and a Hettich centrifuge model ROTOFIX 32A (Kirchlengern, Germany) were used for the preparation of the samples.

### 2.5. Extraction process

**Scheme 1:**
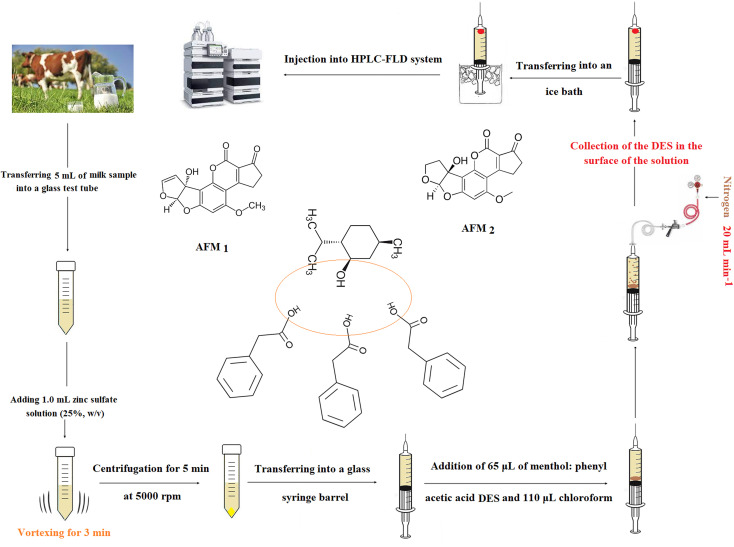
The method steps and structure of the analytes and menthol : phenyl acetic acid DES.

The extraction method was the modified version of perviously published method.^22^ First, a 5 mL raw cow milk sample spiked with the analytes (200 ng L^−1^) or real sample, was taken into a glass test tube and mixed with 1 mL zinc sulfate solution (25%, w/v). The obtained mixture was vortexed for 3 min and consequently the sample proteins were precipitated. After centrifuging (at 5000 rpm for 5 min), the upper phase was transferred into a barrel of a 10 mL glass syringe containing a mixture of 65 μL menthol : phenylacetic acid DES (as the extraction solvent), and 110 μL chloroform (as a density modifier). Subsequently, a needle was used to pierce the septum, and nitrogen gas (99.999%) was flown into the barrel at a flow rate of 20 mL min^−1^ adjusted using a nitrogen mass flow controller. After approximately 3 min, the chloroform content was completely evaporated, and the small droplets of menthol : phenylacetic acid DES were released into the solution and the analytes extracted into its droplets. In the next step, the syringe was placed in an ice bath for 3 min and then a solidified drop was obtained. Finally, this drop was taken into a microtube using a spatula and injected into HPLC-FLD after diluting with the mobile phase till 50 μL. The steps and structure of the analytes and menthol : phenyl acetic acid DES are shown in Scheme 1.

## 3. Results and discussion

### 3.1. Optimization of the extraction parameters

#### 3.1.1. Optimization of zinc sulfate solution concentration and volume

**Fig. 1:**
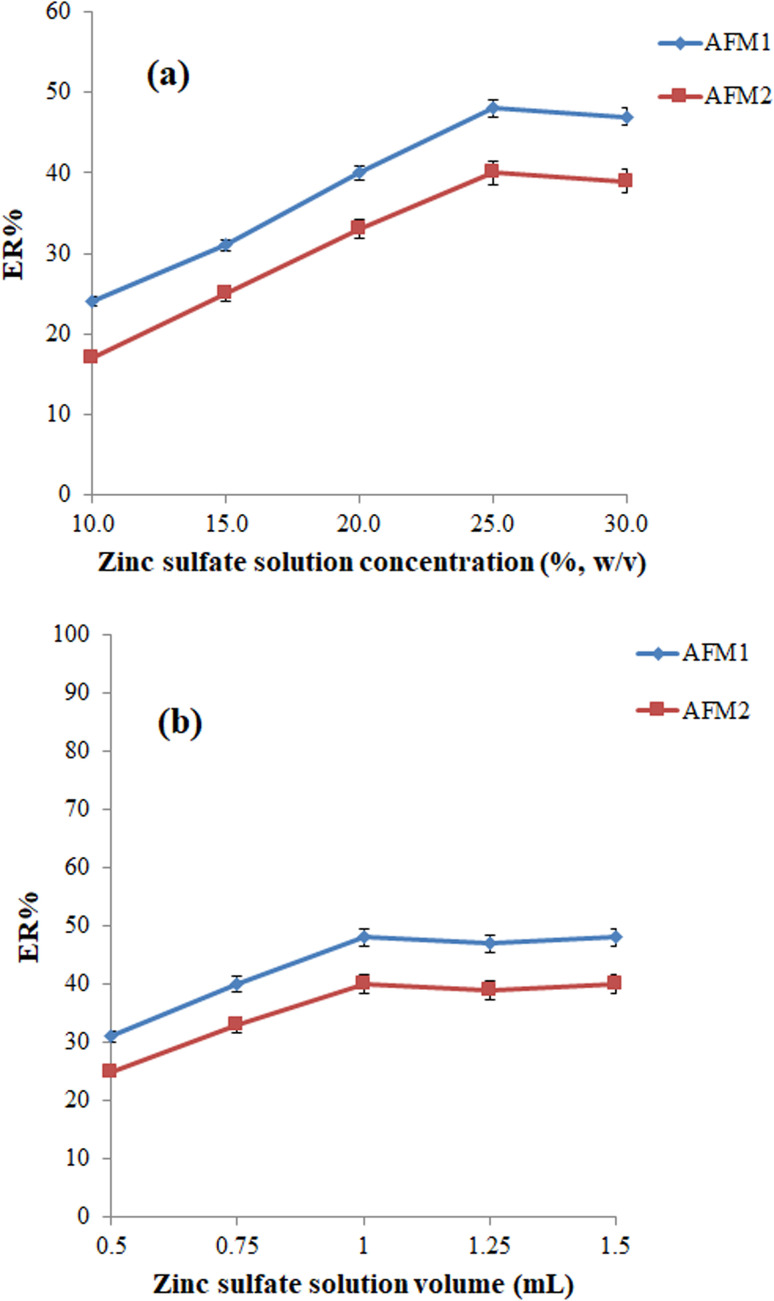
(a). Optimization of zinc sulfate solution concentration. Conditions: sample, 5 mL blank cow milk sample spiked with 200 ng L^−1^ of each analyte; zinc sulfate solution volume, 1 mL; vortexing time, 5 min; centrifuging speed (time), 5000 rpm (5 min); extraction solvent (volume), ChCl : phenylacetic acid DES (80 μL); density modifier (volume), dichloromethane (175 μL); inert gas type (flow rate), nitrogen (25 mL min^−1^). The error bars show the minimum and maximum of three repeated determinations. (b) Optimization of zinc sulfate solution volume. Conditions: the same as those utilized in (a), except 25%, w/v, zinc sulfate solution was used.

Foodstuffs such as milk samples contain high amounts of proteins that can restrict the extraction of analytes. Therefore, it is vital to precipitate proteins from the milk sample to achieve high efficiency. Zinc sulfate was employed to precipitate the proteins. For this purpose, various concentrations (10, 15, 20, 25, and 30%, w/v) of zinc sulfate were employed in the extraction process. Based on the data in Fig. 1a, the ERs of analytes were up to 25% and remained unchanged. Thus, the following experiments were performed using 25%, w/v, of the zinc sulfate solution.

The volume of zinc sulfate solution is another parameter that can affect the efficiency of the protein precipitation step and consequently the efficiency of the extraction process. To optimize this parameter, various studies were performed using 0.50, 0.75, 1.00, 1.25, and 1.50 mL of zinc sulfate solution (25%, w/v). According to the outcomes shown in Fig. 1b, 1.00 is sufficient for the effective precipitation of the proteins.

#### 3.1.2. Optimization of the vortexing time

In this work, the mixture of zinc sulfate solution as the precipitating agent and milk sample was vortexed to increase the contact area between them and effectively precipitate the proteins of the milk sample in the minimum time. For the best results, the mentioned mixture was vortexed for 1 to 5 min. Based on the data (Fig. S1†), the method efficiency increases until 3 min and then reaches constant values. Thus, 3 min was chosen for the next experiments.

#### 3.1.3. Optimization of extraction solvent type and volume

**Fig. 2:**
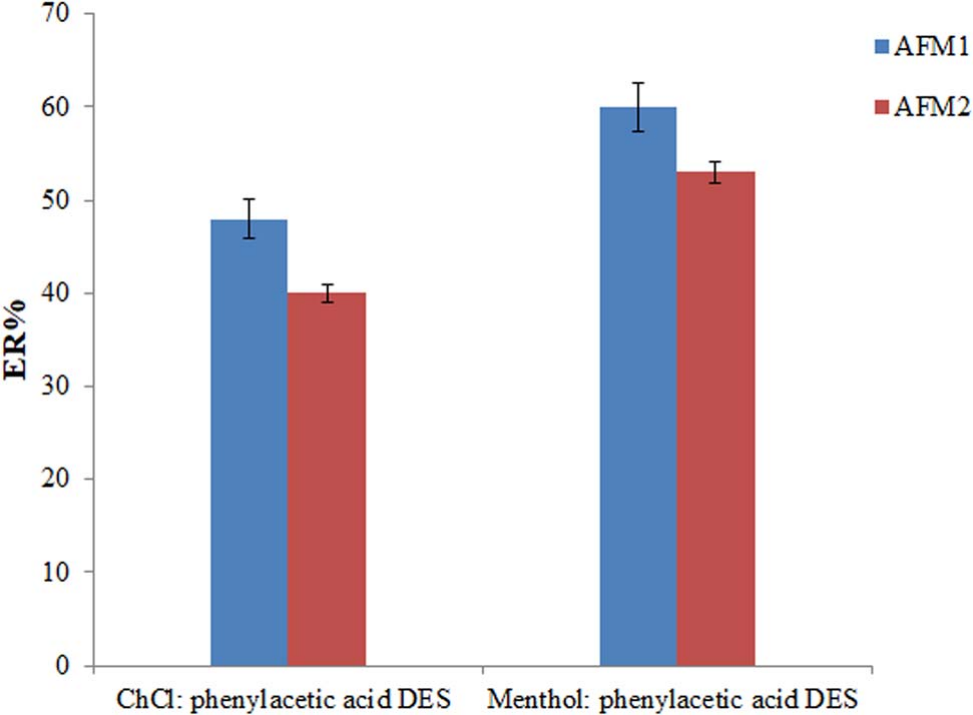
Selection of extraction solvent. Conditions: the same as those utilized in Fig. 1b, except the experiments were done using 1 mL zinc sulfate solution (25%, w/v) and 3 min vortexing time.

Liquids with a lower density than water, a low melting point, low aqueous solubility, and high extraction ability for the analytes are the required conditions for a proper extractant in the offered method. Considering these requirements, a solution containing menthol : phenylacetic acid (65 μL) and ChCl : phenylacetic acid (80 μL) DESs was employed as the extraction solvent in the extraction process (various volumes were used to obtain an equal volume of the collected phase). As can be seen in Fig. 2, the best ERs for analytes were obtained using a menthol : phenylacetic acid as the extraction solvent.

To investigate the effect of menthol : phenylacetic acid volume on the efficiency of the offered method, its volume was varied in the range of 55–80 μL. Referring to the results, the ERs of both analytes increased up to 65 μL and remained constant at the higher volumes. So, the following experiments were performed using 65 μL of menthol : phenylacetic acid DES.

#### 3.1.4. Optimization of ionic strength

In the DLLME-based methods, altering the ionic strength of the sample solution can affect the efficiency of the offered method through salting-out (in which the solubility of the analytes in the aqueous phase decreases and simultaneously enhances their migration into an extraction solvent, which can lead to a higher ER) or salting-in (in which the viscosity of the sample solution was increased as a result of adding a salt) effects. In this step, NaCl at various concentrations (0, 2.5, 5.0, 7.5, and 10%, w/v) was added to the sample solution to determine the optimum concentration. According to the obtained results, the salting-in effect is predominant in this work, and the ERs of both analytes decrease with increasing NaCl concentration. Considering this point, the following studies were performed in the absence of NaCl.

#### 3.1.5. Optimization of the type and volume of the density modifier

**Fig. 3:**
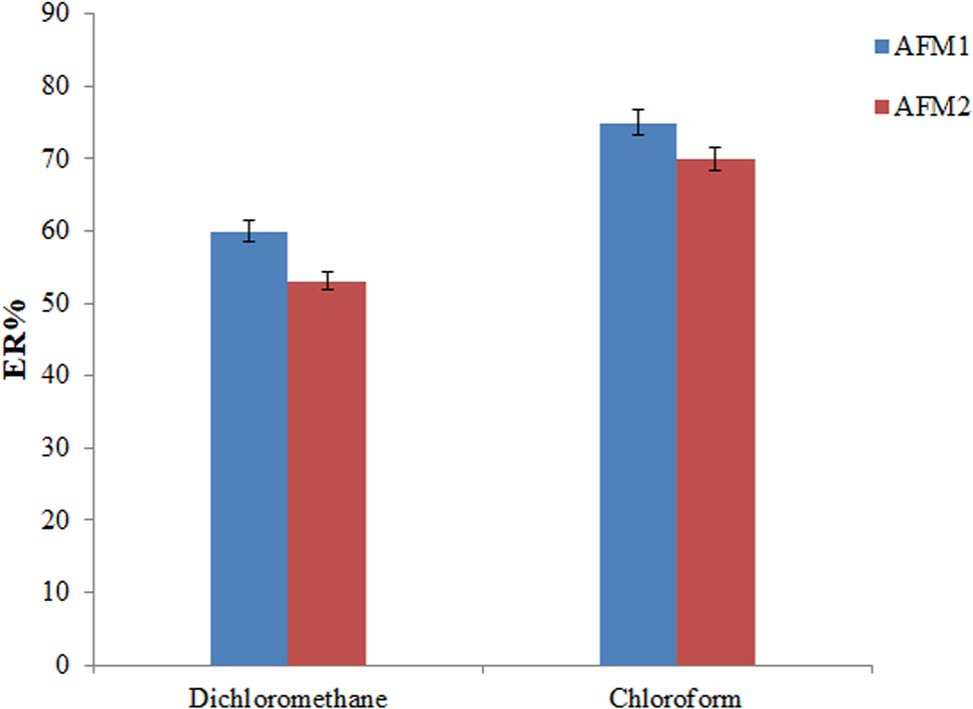
Selection of density modifier. Conditions: the same as those utilized in Fig. 2, except menthol : phenylacetic acid DES (65 μL) was utilized as extractant.

In the offered gas-controlled density-tunable SFOD-DLLME (GCT-DT-SFOD-DLLME) method, a density modifier is required to maintain the extraction solvent at the bottom of the syringe (by increasing density) prior to the bubbling of the gas. For this purpose, dichloromethane (175 μL) and chloroform (110 μL) were tested as density modifier solvents (various volumes were selected due to the different solubility of the mentioned solvents in water). These solvents were selected since they have a density higher than that of water and high vapor pressure (to evaporate in a short time). Referring to the results in Fig. 3, chloroform provides the highest ERs for both analytes (this can be attributed to the slower evaporation of chloroform in comparison with dichloromethane that leads to the release of tiny droplets of the extractant) and was selected as the density modifier solvent in the following experiments.

The volume of chloroform is another parameter that can affect the efficiency of the offered method and the required extraction time. To determine the optimum volume of chloroform, its volume was varied in the range of 110 (this volume was the least possible volume that could maintain the extractant at the bottom of the syringe prior to starting the extraction procedure) to 150 μL (at the intervals of 10 μL). Based on these outcomes, the ERs of the analytes were not altered by increasing the chloroform volume. Therefore, to shorten the required extraction time, 110 μL chloroform was used in the offered extraction procedure.

#### 3.1.6. Optimization of the gas type and flow rate

**Fig. 4:**
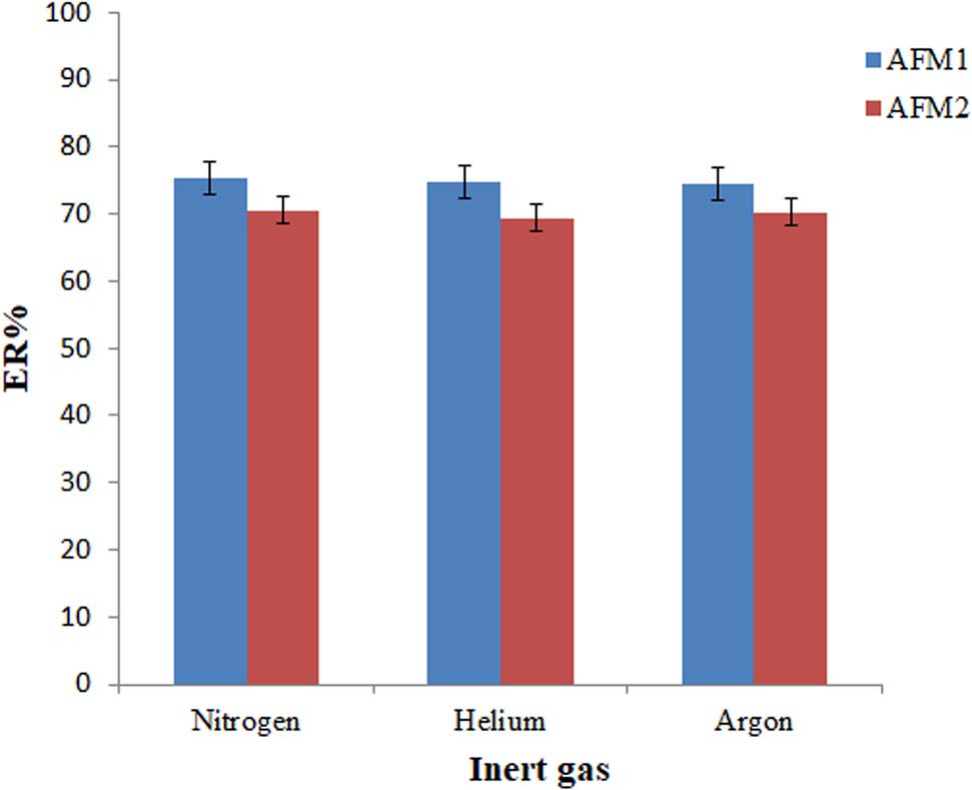
Selection of inert gas type. Conditions: the same as those utilized in Fig. 3, except the 110 μL chloroform was utilized in the extraction process.

The offered GCT-DT-SFOD-DLLME method was started by bubbling in an inert gas (by doing so, the density modifier solvent was completely removed and fine droplets of the extractant were released). Thus, the type and flow rate of the employed gas needed to be optimized. To find a suitable solvent, various experiments were performed in the presence of nitrogen, argon, and helium gases. Considering the results shown in Fig. 4, there is no difference between the utilized gases. Considering this point and regarding the low-cost of nitrogen gas, it was selected to be used in the subsequent experiments.

**Fig. 5:**
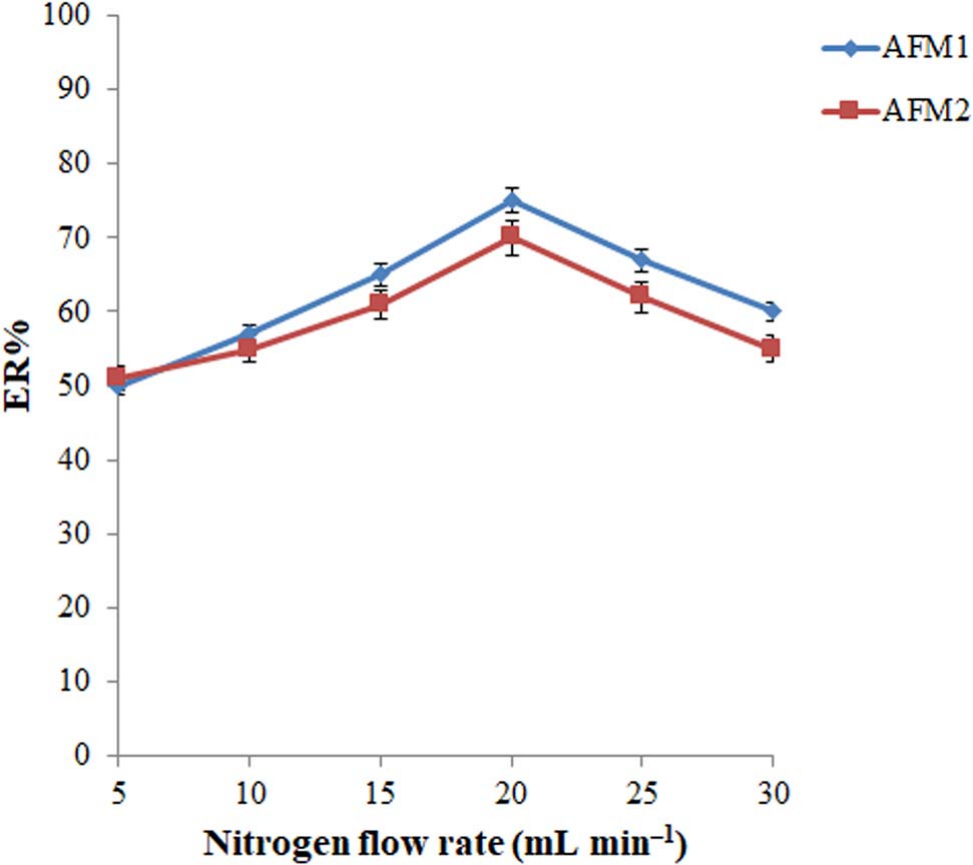
Optimization of inert gas flow rate. Conditions: the same as those utilized in Fig. 4, except nitrogen was used as an inert gas.

The flow rate of nitrogen is another parameter that can affect the required extraction time (the time needed for complete removal of chloroform) and the size of the released extractant droplets that consequently can affect the ERs of the analytes. Considering this point, the gas flow rate was varied in the range of 5 to 30 mL min^−1^ (a bubble flowmeter was employed for this purpose). As can be seen in Fig. 5, the ERs of both analytes increase up to 20 mL min^−1^ and then decrease due to splashing of the extractant on the inner walls of the syringe at higher flow rates and the large size of the released extractant droplets. Therefore, the gas flow rate was adjusted to 20 mL min^−1^ in the subsequent experiments.

### 3.2. Method validation

To assess the analytical performance of the offered method, some figures of merit including a limit of detection (LOD), limit of quantification (LOQ), linear range (LR), coefficient of determination, intra- and inter-day precisions, and ER were evaluated under the best extraction conditions. Based on the data (Table 1), the LODs, considered as signal (S) to noise (N) ratio of 3, were 1.45 and 1.86 ng L^−1^ for AFM_1_ and AFM_2_, respectively. By considering S/N = 10, the LOQs were calculated and data showed that they were 4.83 and 6.21 ng L^−1^ for AFM_1_ and AFM_2_, respectively. The linearity of the offered method obtained from the calibration curves was excellent (*r*^2^ ≥ 0.996) for the wide LRs (4.83–5 × 10^5^ ng L^−1^). ERs 
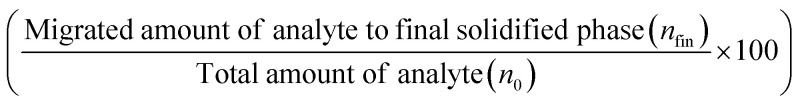
 for AFM_1_ and AFM_2_ were 75% and 70%, respectively. The calibration curves were plotted from the data (peak area) obtained by performing the method on eight solutions *versus* concentration. For evaluation of the repeatability of the method, six solutions spiked at 100 ng L^−1^ were prepared and the obtained peak areas for each analyte after performing the method were recorded. RSD values were calculated on the same day (intra-day) and on four different days (inter-day); they were ≤4.8%.

**Table 1:** Figures of merit of the offered method for the studied AFs

Analyte	LOD^a^ (ng L^−1^)	LOQ^b^ (ng L^−1^)	LR^c^ (ng L^−1^)	*r* ^2^ d	RSD^e^ (%)		ER ± SD^f^
					Intra-day (*n* = 6)	Inter-day (*n* = 4)	
AFM_1_	1.45	4.83	4.83–5 × 10^5^	0.998	3.5	4.2	75 ± 4
AFM_2_	1.86	6.21	6.21–5 × 10^5^	0.996	3.9	4.8	70 ± 2

a Limit of detection (S/N = 3).

b Limit of quantification (S/N = 10).

c Linear range.

d Coefficient of determination.

e Relative standard deviation for intra- and inter-day precisions at a concentration of 100 ng L^−1^ of each analyte.

f Extraction recovery ± standard deviation (*n* = 3).

### 3.3. Analysis of the raw cow milk samples

**Fig. 6:**
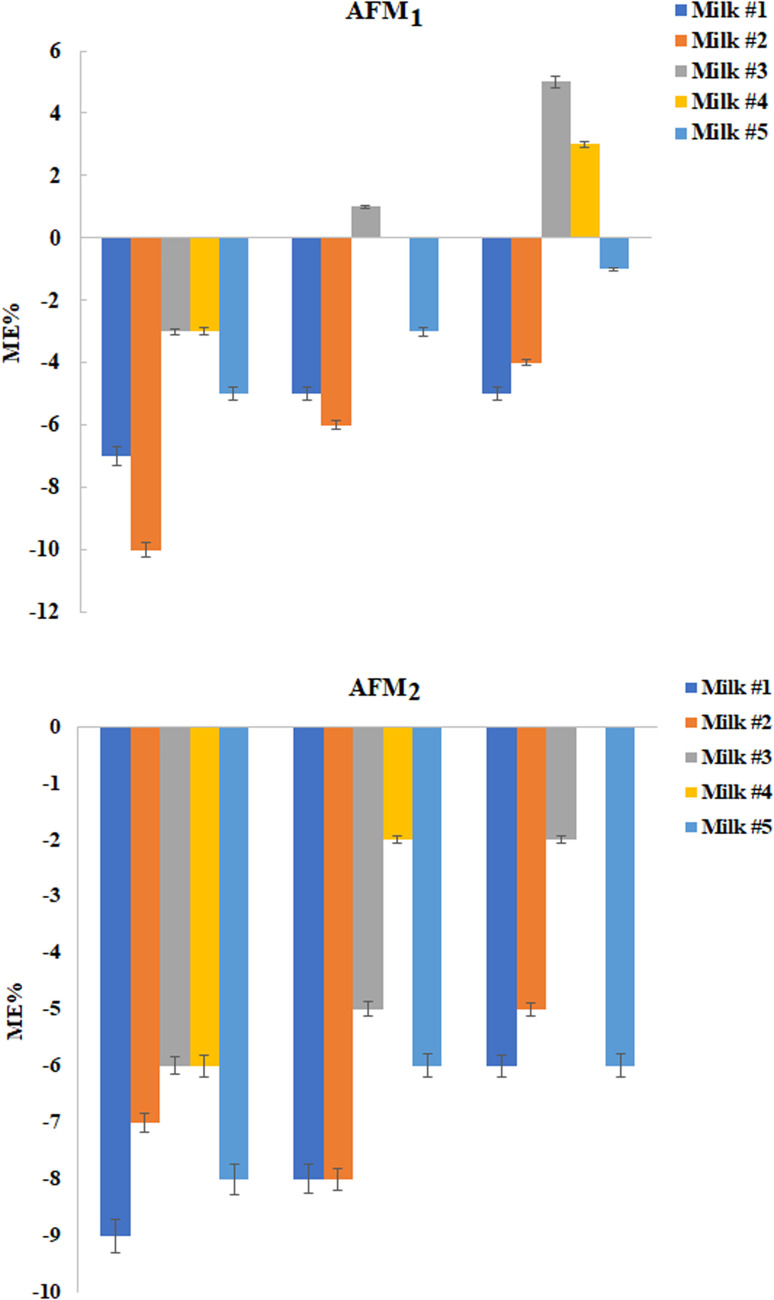
Matrix effect study.

In this step, twenty raw cow milk samples were analyzed for monitoring AFM_1_ and AFM_2_ using the GCT-DT-SFOD-DLLME method under optimized conditions. Based on the outcomes, 9 out of 20 were contaminated with AFM_1_ whose concentration is presented in Table 2. Subsequently, the matrix effect of the raw cow milk samples was examined. As shown in Fig. 6, slight suppression of the analytical signals was obtained at the tested concentrations. The matrix effect (ME%) values were obtained in the range between −20% and +20%, and therefore it can be regarded as insignificant based on the SANTE guidelines.^25^

**Table 2:** Target analytes contents in different samples

Sample	Mean concentration of the analyte (ng L^−1^) ± standard deviation (*n* = 3)	
	AFM_1_	AFM_2_
Milk #1	ND^a^	ND
Milk #2	ND	ND
Milk #3	142 ± 11	ND
Milk #4	96 ± 6	ND
Milk #5	38 ± 2	ND
Milk #6	ND	ND
Milk #7	ND	ND
Milk #8	78 ± 5	ND
Milk #9	ND	ND
Milk #10	ND	ND
Milk #11	ND	ND
Milk #12	ND	ND
Milk #13	103 ± 8	ND
Milk #14	114 ± 12	ND
Milk #15	67 ± 3	ND
Milk #16	132 ± 10	ND
Milk #17	ND	ND
Milk #18	ND	ND
Milk #19	126 ± 9	ND
Milk #20	ND	ND

a Not detected.

### 3.4. Comparison of the method with other approaches

The quantitative data obtained using the offered method (LOD, LOQ, LR, and RSD) were compared with other methods.^26–29^ The details of these data are shown in Table 3. Referring to these results, the LODs and LOQs of the presented method are better than those of the previously reported ones. The RSDs for analytes using the suggested method are better than those from the other methods mentioned. Also, the LRs for the developed method are wider than those of the compared methods. Considering these outcomes, the offered analytical method is suitable for the determination of AFM_1_ and AFM_2_ in raw cow milk samples.

**Table 3:** Comparison of the developed method with the previous ones utilized in the quantification of analytes

Sample	Analyte	RSD^a^ (%)	LOD^b^	LOQ^c^	LR^d^	Method	Ref.
Breast milk	AFM_1_	≤5.9	10 (ng L^−1^)	30 (ng L^−1^)	100–15 × 10^3^ (ng L^−1^)	LLE-LTP-HPLC-FLD^e^	26
Milk	AFM_1_	12.3	60 (ng L^−1^)	210 (ng L^−1^)	210–5 × 10^3^ (ng L^−1^)	HF-LPME-HPLC-MS/MS^f^	27
Peanut, maize and wheat	AFM_1_	8.4	70 (ng kg^−1^)	240 (ng kg^−1^)	10^2^–10^5^ (ng kg^−1^)	SPE-HPLC-MS/MS^g^	28
	AFM_2_	11.4	160 (ng kg^−1^)	520 (ng kg^−1^)	10^2^–10^5^ (ng kg^−1^)		
Milk	AFM_1_	5.28	125.42 (ng kg^−1^)	418.05 (ng kg^−1^)	—	LLE-SPE-HPLC-FLD^h^	29
	AFM_2_	5.71	151.73 (ng kg^−1^)	505.77 (ng kg^−1^)	—		
Milk	AFM_1_		6 (ng L^−1^)	15 (ng L^−1^)	—	Immunoaffinity-HPLC-FLD	24
Milk	AFM_1_	3.5	1.45 (ng L^−1^)	4.83 (ng L^−1^)	4.83–10^6^ (ng L^−1^)	GCT-DT-SFOD-DLLME-HPLC-FLD^i^	Current work
	AFM_2_	3.9	1.86 (ng L^−1^)	6.21 (ng L^−1^)	6.21–10^6^ (ng L^−1^)		

a Relative standard deviation.

b Limit of detection (ng L^−1^).

c Limit of quantification (ng L^−1^).

d Linear range (ng L^−1^).

e Liquid–liquid extraction-low temperature purification-high performance liquid chromatography-fluorescence detector.

f Hollow fiber-liquid phase microextraction-high performance liquid chromatography-tandem mass spectrometry.

g Solid phase extraction-high performance liquid chromatography-tandem mass spectrometry.

h Liquid–liquid extraction-solid phase extraction-high performance liquid chromatography-fluorescence detector.

i Gas-controlled density tunable solidification of floating organic droplet-based dispersive liquid–liquid microextraction-high performance liquid chromatography-fluorescence detector.

## 4. Conclusions

In this study, the concentrations of AFM_1_ and AFM_2_ in raw cow milk samples were determined using GCT-DT-SFOD-DLLME and HPLC-FLD. The removal of the centrifugation step (a time-consuming step) and utilizing DES as an extraction solvent are the main advantages of this method compared with traditional DLLME. According to the validation outcomes, the suggested analytical method offers low LODs and LOQs, low RSDs, high ERs, and an insignificant matrix effect that can verify its suitability for the determination of AFM_1_ and AFM_2_ in raw cow milk samples. Also, referring to the real sample analysis, 45% of the tested raw cow milk samples were contaminated with AFM_1_, which is a warning and needs the attention of related regulatory organizations.

## Conflicts of interest

There are no conflicts to declare.

## Supplementary Material

RA-014-D3RA04149B-s001
