# Supplementary material for: HPLC-FLD determination of aflatoxins M1 and M2 in raw cow milk samples using in-syringe gas-controlled density tunable solidification of a floating organic droplet-based dispersive liquid–liquid microextraction method
Source: RSC Adv. 2024 Feb 8;14(8):5077–84. doi: 10.1039/d3ra04149b (PMC10851050; doi:10.1039/d3ra04149b)
Supplement: RA-014-D3RA04149B-s001 [file RA-014-D3RA04149B-s001.pdf]

Electronic Supplementary Material (ESI) for RSC Advances.
This journal is © The Royal Society of Chemistry 2024

Supplementary data

HPLC–FLD determination of aflatoxins M_1 and M_2 in raw cow milk samples using in–syringe gas–controlled density tunable solidification of floating organic droplet–based dispersive liquid–liquid microextraction method

Maede Rabie[1], Mohammadhosein Movassaghghazani[2,*], Mohammad Reza Afshar Mogaddam [3,4]

[1] Faculty of Veterinary Medicine, Shabestar Branch, Islamic Azad University, Shabestar, Iran

[2] Department of Food Hygiene and Quality Control, Faculty of Veterinary Medicine, Shabestar Branch, Islamic Azad University, Shabestar, Iran

[3] Food and Drug Safety Research Center, Tabriz University of Medical Sciences, Tabriz, Iran

[4]Pharmaceutical Analysis Research Center, Tabriz University of Medical Sciences, Tabriz, Iran

Corresponding author: Dr. M. Movassaghghazani

Email: mh.movassagh@iau.ac.ir; drmhmg@gmail.com

Phone: +989143010292

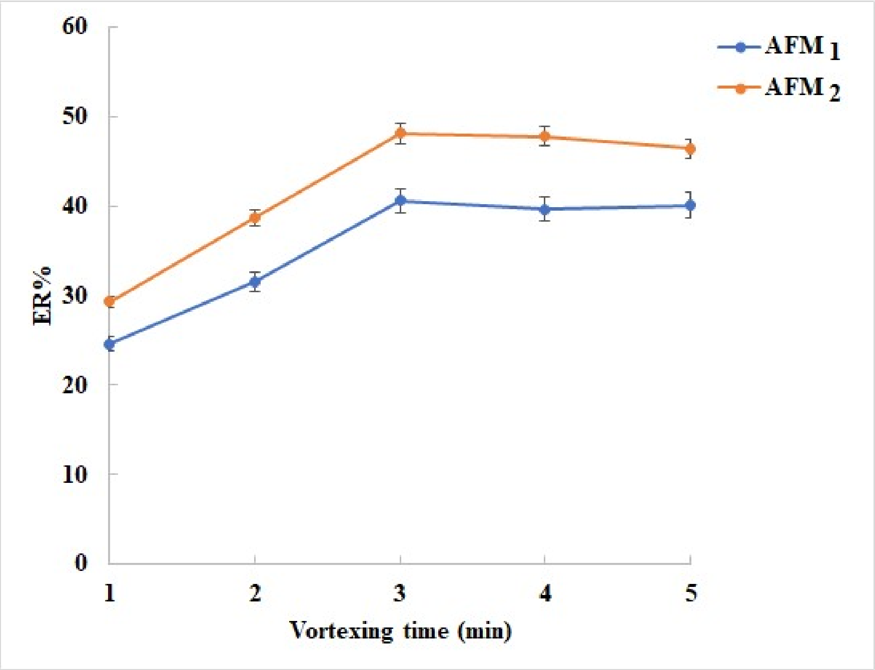

Fig. S1. Optimization of vortexing time

Conditions: sample, 5 mL blank raw cow milk sample spiked with 200 ng L^{-1} of each analyte; zinc sulfate solution concentration (volume), 25% w/v (1 mL); centrifuging speed (time), 5000 rpm (5 min); extraction solvent (volume), ChCl: phenylacetic acid DES (80 μL); density modifier (volume), dichloromethane (175 μL); inert gas type (flow rate), nitrogen (25 mL min^{-1}). The error bars show the minimum and maximum of three repeated determinations.
